# A Superparaelectric State in Relaxor Ferroelectric (Sr,Bi)TiO_3_-Bi(Mg,Ti)O_3_-Modified BaTiO_3_ Ceramics to Achieve High Energy Storage Performance

**DOI:** 10.3390/ma17020426

**Published:** 2024-01-15

**Authors:** Il-Ryeol Yoo, Seong-Hui Choi, Je-Yeon Park, Min-Seok Kim, Arun Kumar Yadav, Kyung-Hoon Cho

**Affiliations:** School of Materials Science and Engineering, Kumoh National Institute of Technology, Gumi 39177, Republic of Korea; yiy1204@kumoh.ac.kr (I.-R.Y.); shchoi7@kumoh.ac.kr (S.-H.C.); wpdus7878@kumoh.ac.kr (J.-Y.P.); king611@kumoh.ac.kr (M.-S.K.); arunme40016@gmail.com (A.K.Y.)

**Keywords:** dielectric ceramic capacitor, relaxor ferroelectric, superparaelectric, energy storage density, BaTiO_3_

## Abstract

Dielectric ceramic capacitors are highly regarded for their rapid charge–discharge, high power density, and cyclability in various advanced applications. However, their relatively low energy storage density has prompted intensive research aiming at developing materials with a higher energy density. To enhance energy storage properties, research has focused on modifying ferroelectric materials to induce relaxor ferroelectricity. The present study aims to induce a superparaelectric (SPE) state in relaxor ferroelectrics near room temperature by altering BaTiO_3_ ferroelectric ceramics using the (Sr,Bi)TiO_3_-Bi(Mg_0.5_Ti_0.5_)O_3_ system ((1−x)BT-x(SBT-BMT)). X-ray diffraction and Raman spectroscopy analysis demonstrated a shift in the crystal structure from tetragonal to cubic with an increasing x content. Notably, the compositions (except x = 0.1) satisfied the criteria for the SPE state manifestation near room temperature. The x = 0.2 specimen displayed characteristics at the boundary between the relaxor ferroelectric and SPE phases, while x ≥ 0.3 specimens exhibited increased SPE state fractions. Despite reduced maximum polarization, x ≥ 0.3 specimens showcased impressive energy storage capabilities, attributed to the enhanced SPE state, especially for x = 0.3, with impressive characteristics: a recoverable energy density (*W_rec_*) of ~1.12 J/cm^3^ and efficiency (*η*) of ~94% at 170 kV/cm applied field. The good stability after the charge–discharge cycles reinforces the significance of the SPE phase in augmenting energy storage in relaxor ferroelectric materials, suggesting potential applications in high-energy density storage devices.

## 1. Introduction

Among various energy storage devices, dielectric ceramic capacitors exhibit advantages in advanced fields such as defense, healthcare, and smart mobility due to their rapid charge–discharge, high power density, and excellent cyclability [1,2,3]. However, the relatively low energy storage density of dielectric capacitors limits their practical applications, prompting extensive research aiming at developing dielectric ceramic materials with a higher energy density [4,5,6,7,8]. The energy storage characteristics of dielectrics depend on the changes in polarization, induced by an external electric field, and are commonly expressed by the following equations:

(1) $$W=\int_{0}^{P_{max}}EdP$$

(2) $$W_{rec}=\int_{P_{r}}^{P_{max}}EdP$$

(3) $$\eta =\frac{W_{rec}}{W}\times 100\%$$

Here, *W* represents the charging energy storage density, *W_rec_* stands for the recoverable energy storage density (discharge energy storage density), *η* denotes the efficiency, and *P_max_* and *P_r_*, respectively, indicate the maximum polarization during the charging process and the remnant polarization during the discharge process. To achieve a high energy density and high efficiency, a high *P_max_*, a high dielectric breakdown strength, and a small hysteresis area on the polarization-electric field (P-E) curve are required.

Research aimed at enhancing the energy storage properties of dielectric ceramics has primarily progressed towards inducing relaxor ferroelectricity through compositional modifications of ferroelectric materials [9,10]. While normal ferroelectrics possess high *P_max_* values, their large *P_r_* values lead to lower *W_rec_* and *η*. By doping various ions, it is possible to transform long-range ordered microscale ferroelectric domains into short-range ordered nanoscale domains, thereby inducing relaxor ferroelectricity [11,12,13]. The reduction in domain size and weakening inter-domain coupling reduce the domain switching energy barriers, minimizing hysteresis and consequently increasing *W_rec_* and *η* [14,15,16,17,18].

In relaxor ferroelectrics, there are three characteristic temperatures that are sequentially observed at high temperatures: (1) the Burns temperature (*T_B_*), where nanodomains begin to form, (2) *T_max_*, where nanodomains grow, reaching the maximum dielectric permittivity, and (3) the freezing temperature (*T_f_*), where nanodomains freeze, and which represents irreversible ferroelectricity [19]. Previous studies on relaxor ferroelectrics mainly evaluated the energy storage performance within the temperature range of *T_f_* < *T* < *T_max_* [4,5]. In this case, although high *P_max_* values can be achieved, the presence of large hysteresis results in low *W_rec_* and *η*. To simultaneously achieve high *W_rec_* and *η*, attention needs to be given to the reported superparaelectric (SPE) relaxor ferroelectrics [7,8,9]. The SPE state occurs within the temperature range of *T_max_* < *T* < *T_B_*. In this temperature range, the size of nanodomains significantly decreases, and inter-domain coupling weakens drastically, resulting in extremely slim hysteresis due to significantly reduced domain switching energy barriers [16,17,18].

In this study, we aimed to induce an SPE state near room temperature by compositional modification of BaTiO_3_ (BT) ferroelectric ceramics. To harness robust relaxor ferroelectric properties from BT, which exhibits a high ferroelectric-to-paraelectric transition temperature (~120 °C), we opted for compositional modification using the (Sr,Bi)TiO_3_-Bi(Mg_0.5_Ti_0.5_)O_3_ system. The (Sr,Bi)TiO_3_ (SBT) ceramics exhibit relaxor ferroelectric behavior, characterized by *Bi*^3+^ ions partially substituting the A-site of cubic SrTiO_3_ ceramics. The introduction of *Bi*^3+^ ions aids in creating an off-center ion on the A-site, attributed to the 6s^2^ lone pair nature of *Bi*^3+^. This substitution of *Bi*^3+^ for *Sr*^2+^ at the A-site results in a charge imbalance, leading to the generation of *Sr* vacancies and subsequent defects within the system. These defect centers, along with off-center *Bi*-ions, induce dipoles and local random fields. These local random fields play a crucial role in the formation of polar nanoregions, consequently manifesting relaxor ferroelectric behavior [20,21,22,23,24,25,26]. Research on energy storage through compositional modification of SBT ceramics has also been extensively reported [26,27,28,29]. Moreover, it has been reported that incorporating Bi(Mg_0.5_Ti_0.5_)O_3_ (BMT) at levels exceeding 5 mol% into BT ceramics induces a pseudo-cubic state [30]. Therefore, we designed the (1−x)BT-x(SBT-BMT) system to explore compositions that satisfy the condition of *T_max_* < *T* < *T_B_*. We then investigated the structural, dielectric, ferroelectric, and energy storage properties concerning variations in x.

## 2. Materials and Methods

The (1−x)BaTiO_3_-x(0.75(Sr_0.88_Bi_0.08_)TiO_3_-0.25Bi(Mg_0.5_Ti_0.5_)O_3_) ((1−x)BT-x(SBT-BMT)) ceramics with x = 0.1, 0.2, 0.3, and 0.4 were prepared using solid-state synthesis. Raw material powders including BaCO_3_ (99.95%, Kojundo Korea Co., Uiwang-si, Korea), TiO_2_ (99.99%, Kojundo Korea Co., Uiwang-si, Korea), SrCO_3_ (99.9%, Kojundo Korea Co., Uiwang-si, Korea), Bi_2_O_3_ (99.9%, Kojundo Korea Co., Uiwang-si, Korea), and MgO (99.9%, Kojundo Korea Co., Uiwang-si, Korea) were utilized for the synthesis process. The raw powders, weighed according to each composition, were placed into a nylon container containing zirconia balls (YSZ, SciLab, Seoul, Korea) and anhydrous ethanol (99.9%, Samchun Pure Chemical Co., Seoul, Korea). These were subjected to a typical ball milling (~200 rotations per minute) process for 12 h to undergo primary grinding and mixing. After mixing, the slurries were dried and then calcined at 900 °C for 2 h. The calcined powders underwent another milling process using the same ball milling procedure for 24 h. Then, the dried powders were subjected to a uniaxial pressure of 100 MPa to shape them into discs (diameter: 10 mm, thickness: 1.5 mm). Subsequently, they were sintered at temperatures ranging from 1200 to 1275 °C for 2 h at a heating rate of 4 °C/min. Finally, they were cooled down to room temperature inside the furnace. Specifically, for x = 0.1, the sintering temperature was 1275 °C; for x = 0.2, it was 1250 °C; for x = 0.3, it was 1225 °C; and for x = 0.4, it was 1200 °C. The corresponding relative densities of these samples were found to be 94% (for x = 0.1), 95% (for x = 0.2), 96% (for x = 0.3), and 95% (for x = 0.4). To measure the electrical characteristics of the sintered specimens, Ag paste was applied to both sides. The specimens were then heated at a rate of 4 °C/min until reaching 700 °C, where the Ag electrodes were sintered for 1 h.

The phase and structural analysis of the (1−x)BT-x(SBT-BMT) ceramics was conducted by X-ray diffraction (XRD) (SmartLab, Rigaku, Tokyo, Japan) with Cu-Kα radiation (λ = 1.54056 Å). The XRD data were obtained in the *2θ* range of 15–80° at a scanning rate of 1°/min with a 0.02° step. Raman spectroscopy (System1000, Renishaw, Wotton-under-Edge, UK) was used to analyze the vibrational properties, with a ± 0.001 cm^−1^ error limit. Scanning electron microscope (SEM) (JSM-6500F, JEOL, Tokyo, Japan) was utilized to see the surface morphology of the sintered samples with a 15 kV acceleration voltage. Temperature-dependent dielectric tests were conducted with impedance analyzer (IM3570, Hioki, Ueda, Japan) at frequencies of 10 kHz, 50 kHz, and 100 kHz, using a spring-loaded, Pt-based contact mechanism capable of withstanding temperatures up to 600 °C. The tests were performed with a 1 V oscillation potential and a heating rate of 3 °C/min in a rectangular muffle furnace, with a ±2 °C error value. Additionally, another temperature sensor (with a ±1 °C error) was positioned close to the sample during measurements near the sample stage. The corresponding values were recorded using automatic LabVIEW 2015 SP1 software connected to a computer via a cable.

The ferroelectric test was performed using a PK-CPE1801 ferroelectric test system (PolyK Technologies, Philipsburg, PA, USA), equipped with a PolyK sample stage made of stainless steel and aluminum. During the measurement, the sample stage was immersed in silicon oil.

## 3. Results and Discussion

**Figure 1:**
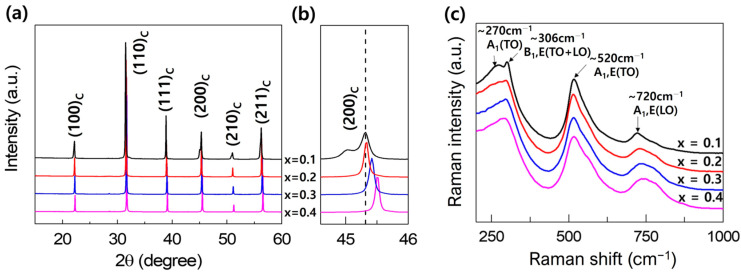
(**a**) XRD patterns of sintered (1−x)BT-x(SBT-BMT) ceramics. (**b**) Enlarged view of the XRD (200) peaks. (**c**) Raman spectra of (1−x)BT-x(SBT-BMT) ceramics.

Figure 1a depicts the XRD patterns of the sintered (1−x)BT-x(SBT-BMT) ceramics. All samples exhibited a perovskite structure without any detectable secondary phases within the XRD limits. To elucidate the peak shape, the *2θ* range between 44.6 and 46° is magnified in Figure 1b. The diffraction peak near 45° split into two peaks for the x = 0.1 specimen, indicating a tetragonal phase (space group: *P*4*mm*, JCPDS No. 05-0626) [31]. The diffraction peaks merged into a single peak for the compositions with x ≥ 0.2, indicating a cubic phase (space group:$\;Pm\bar{3}m$, JCPDS No. 31-0174) [32]. Additionally, the diffraction peaks exhibited a shift towards higher *2θ* values with increasing x (Figure 1b), suggesting a lattice contraction due to the substitution of elements with smaller ionic radii by the perovskite A-site (*r*(*Ba*^2+^): 1.61 Å, *r*(*Sr*^2+^): 1.44 Å, and *r*(*Bi*^3+^): 1.45 Å [33,34]. To evaluate the lattice parameters as a function of x, Rietveld refinement was performed using the Fullprof software package version 7.95. The x = 0.1 composition fit well with the *P*4*mm* space group, whereas the compositions with x ≥ 0.2 fit well with the $Pm\bar{3}m$ space group. The calculated lattice parameters are presented in Table 1. Additionally, Raman spectroscopy was conducted in the range of 100 to 1000 cm^−1^ to investigate the vibrational properties of the (1−x)BT-x(SBT-BMT) ceramics, as shown in Figure 1c. Raman modes were evidenced at 270, 306, 520, and 720 cm^−1^. BaTiO_3_ exhibits a tetragonal (P4mm) structure at room temperature, which is characterized by a sharp mode at 306 cm^−1^ along with asymmetric and broad modes at 270, 520, and 720 cm^−1^ in its Raman spectrum [35,36,37]. Furthermore, above 120 °C, BaTiO_3_ undergoes a phase transition to the cubic phase ($Pm\bar{3}m$), resulting in the weakening and broadening of the 306 cm^−1^ mode, along with further broadening of the 270, 520, and 720 cm^−1^ modes [38,39,40]. For the x = 0.1 specimen, a sharp mode was observed at 306 cm^−1^. However, for the compositions with x ≥ 0.2, the intensity of the 306 cm^−1^ mode decreased and broadened. Additionally, the modes at 270, 520, and 720 cm^−1^ also became broader, indicating a cubic phase. The modes at 270, 306, 520, and 720 cm^−1^ were identified as A_1_(TO), B_1_,E(TO + LO), A_1_,E(TO), and A_1_,E(LO), respectively, as presented in Figure 1c. These nomenclatures are described in detail in the reported results [35,41]. The XRD and Raman spectra results in Figure 1 both indicate that SBT-BMT was incorporated into the BT lattice successfully.

**Figure 2:**
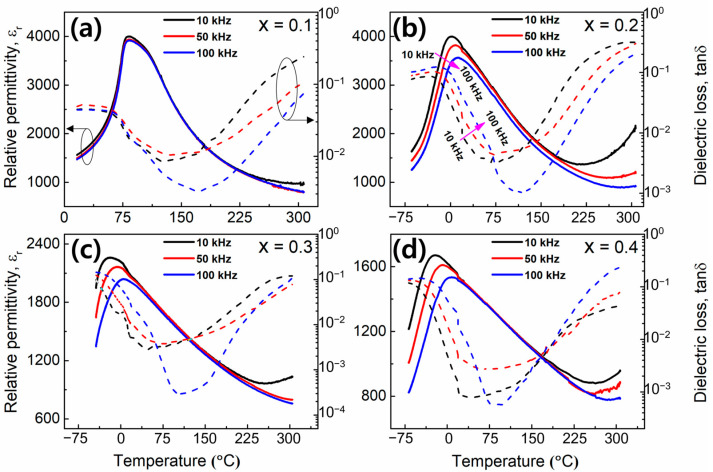
Relative dielectric permittivity (*ε_r_*) and loss tangent (*tan δ*) versus temperature for (1−x)BT-x(SBT-BMT) ceramics, where (**a**) x = 0.1, (**b**) x = 0.2, (**c**) x = 0.3, and (**d**) x = 0.4. The y-axis of *tan δ* versus temperature is presented in logarithmic scale to enhance data visualization.

Figure 2a–d illustrate the variation in the relative dielectric permittivity (*ε_r_*) and dielectric loss tangent (*tan δ*) of the (1−x)BT-x(SBT-BMT) ceramics at different fixed frequencies (10 kHz, 50 kHz, and 100 kHz) with increasing temperature. For x = 0.1, the measuring temperature ranges from 16 to 306 °C, while for x ≥ 0.2, it ranges from −68 to 306 °C. The x = 0.1 ceramic exhibited a ferroelectric (tetragonal) to paraelectric (cubic) phase transition temperature at 83 °C (Figure 2a), in contrast to the pure BaTiO_3_, which underwent the phase transition at 120 °C. Furthermore, as x increased, *T_max_* (the temperature at which the maximum relative dielectric permittivity (*ε_m_*) occurs) dramatically decreased from 83 °C (for x = 0.1) to <2 °C (for x = 0.4, Figure 2d), and the relative dielectric permittivity curve flattened and widened, demonstrating relaxor characteristics [42]. The decrease in *T_max_* towards lower temperatures is associated with a reduction in the tetragonality factor (*c/a* ratio) due to the substitution of SBT-BMT into the BT lattice, as indicated by XRD and Raman spectroscopy [43,44]. Furthermore, the introduction of SBT-BMT into the BT host lattice increases the site disorder and charge fluctuation, thereby reducing the polar strength of TiO_6_ octahedra and resulting in a decrease in *T_max_* with increasing x. For the compositions with x ≥ 0.2, both *ε_r_* and *tan δ* exhibited frequency dispersions in the temperature range from the starting temperature to near *T_max_*. These frequency dispersions exhibited opposite trends in *ε_r_* and *tan δ* (as indicated by the arrows in Figure 2b), suggesting relaxor features in the materials [45,46]. The dielectric dispersion behavior of the (1−x)BT-x(SBT-BMT) ceramics was investigated using the modified Curie–Weiss law as described in Equation (4),

(4) $$\frac{1}{\varepsilon_{r}}-\frac{1}{\varepsilon_{m}}=\frac{(T-T_{max}{)}^{\gamma }}{C}(\gamma :degree\;of\;diffuseness,C:constant)$$

where *γ* represents the degree of diffuseness, ranging between 1 (normal ferroelectric) and 2 (ideal relaxor ferroelectric) [47].

**Figure 3:**
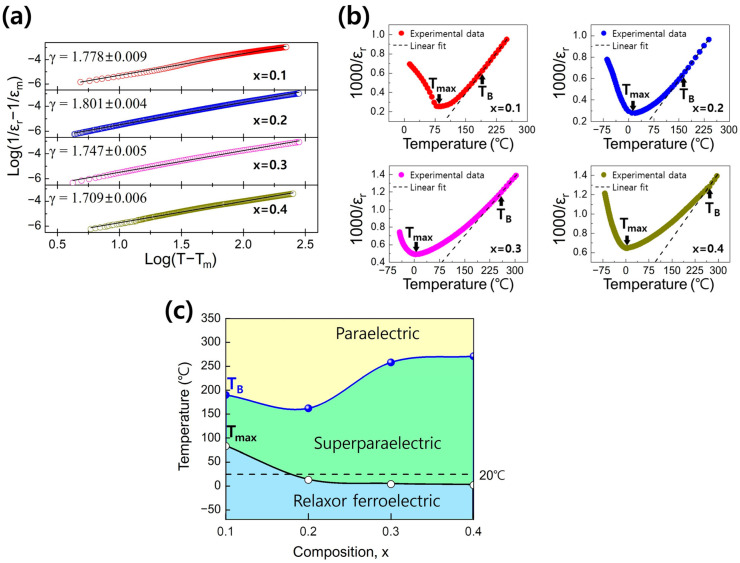
Dielectric characteristics of (1−x)BT-x(SBT-BMT) ceramics: (**a**) log(1/*ε_r_* − 1/*ε_m_*) vs. log(*T* − *T_m_*) curves, (**b**) 1000/*ε_r_* vs. *T* curves, and (**c**) phase diagram based on *T_max_* and *T_B_*.

The *γ* values for all compositions were calculated to be above 1.7 (Figure 3a), which aligned well with the relaxor characteristics shown in Figure 2. The *γ* value increased to 1.8 until x = 0.2. However, for x ≥ 0.3, *γ* slightly decreased to 1.709. This decrease is attributed to the weakening of the ferroelectricity in the relaxor ferroelectric material [48,49]. The weakening of ferroelectricity in relaxor ferroelectric materials is observed during the transition from a normal relaxor ferroelectric state to the superparaelectric (SPE) state [6]. In relaxor ferroelectric materials in the SPE state, a higher energy storage efficiency can be achieved compared to the normal relaxor ferroelectric state due to an increase in the nonpolar phase and a decrease in the ferroelectric domain fraction [7,8,9,16,17,18].

We investigated the *T_max_* and *T_B_* of the (1−x)BT-x(SBT-BMT) ceramics to identify compositions representing the SPE state. *T_B_* refers to the onset temperature that satisfies the Curie–Weiss law in Equation (5), and in Figure 3b, we depict the *T_max_* and *T_B_* of the (1−x)BT-x(SBT-BMT) ceramics.

(5) $$\varepsilon_{r}=\frac{C}{T-T_{0}}(T_{0}:Curie-Weiss\;temperature)$$

Except for x = 0.1, all compositions satisfy the condition of *T_max_* < *T* < *T_B_* near room temperature, indicating that these compositions represent the SPE state. Based on the results in Figure 3b, we illustrate the composition vs. temperature phase diagram for normal relaxor ferroelectric, SPE, and paraelectric phases in Figure 3c. As x increases, the temperature range of the SPE phase expands, and it is confirmed that specimens x = 0.3 and 0.4 are clearly in the SPE state near room temperature.

**Figure 4:**
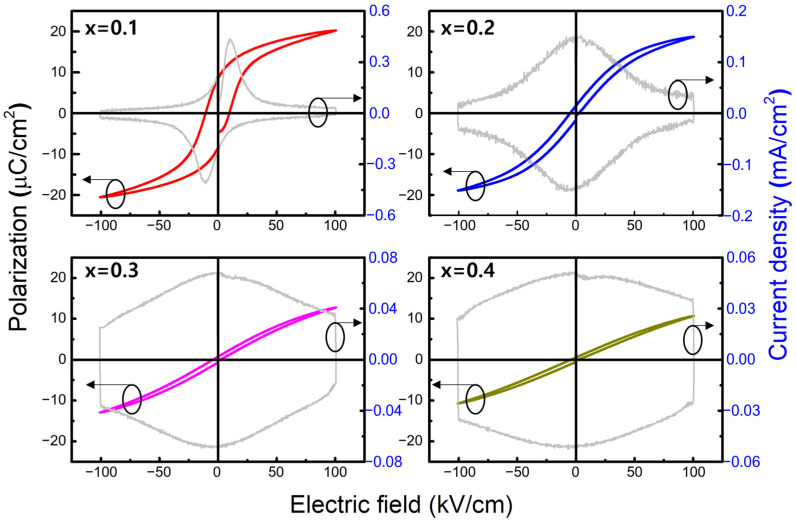
Polarization and current density versus electric field (P-E and J-E) curves of (1−x)BT-x(SBT-BMT) ceramics at a frequency of 10 Hz and an applied field of 100 kV/cm.

Figure 4 represents the room temperature ferroelectric properties of the (1−x)BT-x(SBT-BMT) ceramics measured at a frequency of 10 Hz. At x = 0.1, the P-E and J-E curves of typical ferroelectric materials were obtained, indicating that the x = 0.1 specimen was in the state of *T* < *T_f_*. However, at x = 0.2, the specimen exhibited broad peaks in the J-E curve, observed around *E* = 0 kV/cm, and a slim P-E curve, indicating the typical characteristics of the relaxor ferroelectric material. With an increase in x, the P-E curve became slimmer, and the peaks in the J-E curve broadened further, showing an increase in the squareness of the J-E curve. The increased squareness of the J-E curve indicated the enhancement of paraelectric characteristics [50,51]. Given the phase diagram depicted in Figure 3c, the x = 0.2 specimen was lying near the boundary between the relaxor ferroelectric phase and the SPE phase around room temperature. Therefore, its relative fraction of the SPE state could be small. However, for compositions with x ≥ 0.3, the phase boundary temperature decreased sufficiently below room temperature, suggesting a stronger enhancement of the SPE state in these compositions.

**Figure 5:**
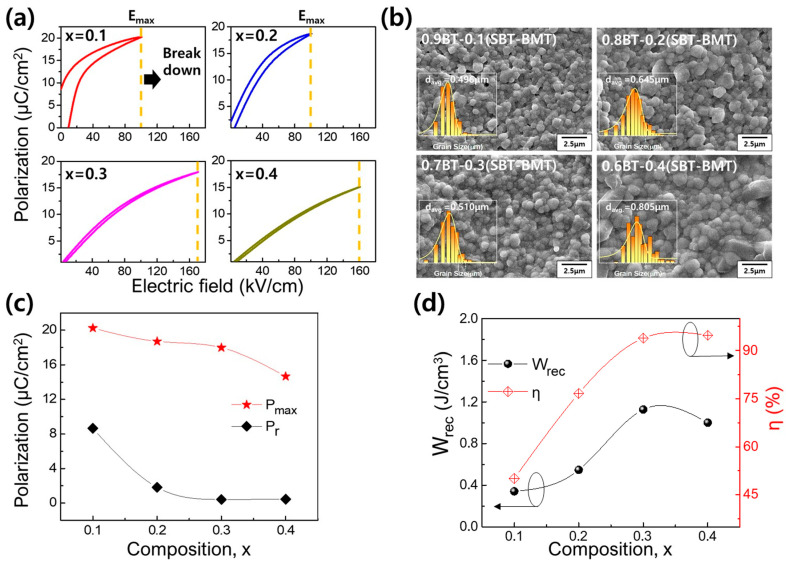
Energy storage characteristics of (1−x)BT-x(SBT-BMT) ceramics: (**a**) room temperature unipolar P-E curves at 10 Hz, (**b**) SEM microstructure (insert: grain size distribution), (**c**) *P_max_* and *P_r_*, and (**d**) *W_rec_* and *η* as a function of x measured at *E_max_*.

A high dielectric breakdown strength (DBS) is crucial in ensuring a high energy storage density. As seen in Figure 5a, the maximum available electric field (*E_max_*) of the (1−x)BT-x(SBT-BMT) ceramics was significantly higher for specimens at x = 0.3 and 0.4 compared to those at x = 0.1 and 0.2. In each composition, the *E_max_* value was deliberately set to be lower than the DBS. In the case of the x = 0.3 composition, five specimens were tested, resulting in *E_max_* values of 168 kV/cm (first specimen), 170 kV/cm (second specimen), 172 kV/cm (third specimen), 170 kV/cm (fourth specimen), and 176 kV/cm (fifth specimen). The corresponding DBS value, determined using the Weibull distribution function, was approximately 172 kV/cm. Consequently, the *E_max_* value of 170 kV/cm, which was lower than the DBS, was used to evaluate the energy storage properties of the x = 0.3 specimen. The DBS values for the other compositions were determined as follows: 102 kV/cm (for x = 0.1), 104 kV/cm (for x = 0.2), and 163 kV/cm (for x = 0.4). Subsequently, the *E_max_* values of 100 kV/cm (for x = 0.1 and x = 0.2) and 160 kV/cm (for x = 0.4) were selected to assess their energy density properties in Figure 5. Despite the significantly larger grain size in the x = 0.4 specimen compared to that in the x = 0.1 specimen (Figure 5b), the notably higher *E_max_* value exhibited by the x = 0.4 specimen indicates a substantial dependence of the dielectric breakdown strength of the (1−x)BT-x(SBT-BMT) ceramics on the fraction of the SPE state. The high *P_max_* and low *P_r_* are also essential for achieving a high energy storage density. As observed in Figure 5c, *P_max_* shows a gradual decrease with increasing x, indicating that the emergence of the SPE state leads to a reduction in the fraction of polar nanoregions (PNRs). This reduction weakens the mutual interaction between ferroelectric domains [16,17,18,52,53]. However, the increase in the SPE phase and the decrease in PNRs had a more significant effect of sharply reducing the *P_r_* (Figure 5c). Despite the decrease in *P_max_*, the remarkable increase in dielectric breakdown strength and the sharp reduction in *P_r_* resulted in compositions with x ≥ 0.3 exhibiting excellent energy storage performance, with *W_rec_* > 1 J/cm^3^ and *η* > 93% (Figure 5d).

**Figure 6:**
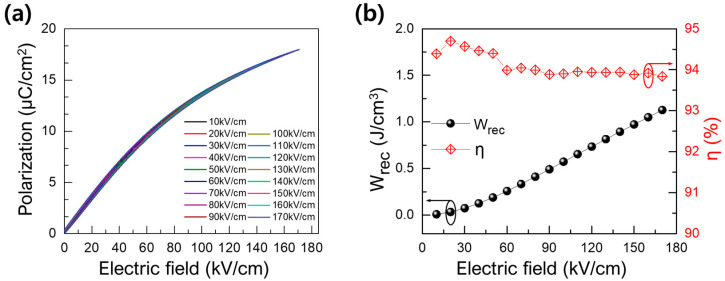
(**a**) Unipolar P-E curves of the 0.7BT-0.3(SBT-BMT) ceramic under different electric fields at a frequency of 10 Hz and (**b**) the calculated *W_rec_* and *η* of the 0.7BT-0.3(SBT-BMT) ceramic as a function of applied electric field.

Figure 6 represents the room temperature energy storage characteristics of the x = 0.3 (0.7BT-0.3(SBT-BMT)) specimen according to the electric field strength at a frequency of 10 Hz. Very slim unipolar P-E curves were observed at all electric field conditions (Figure 6a), resulting in a consistently high *η* of about 94% at all electric field conditions (Figure 6b). *W_rec_* increased linearly with the increase in the electric field, showing a high value of 1.12 J/cm^3^ in an electric field of 170 kV/cm. Table 2 was designed to compare the energy storage properties of the 0.7BT-0.3(SBT-BMT) ceramic with those reported for other BT-based ceramics. From a practical application perspective, it is reasonable to regard both energy density and efficiency as equally crucial factors for high-energy-density capacitors. While the achieved energy density of the 0.7BT-0.3(SBT-BMT) ceramic is comparable to most reported values in Table 2, with only a few exceptions, its efficiency stands out notably by being particularly competitive.

**Figure 7:**
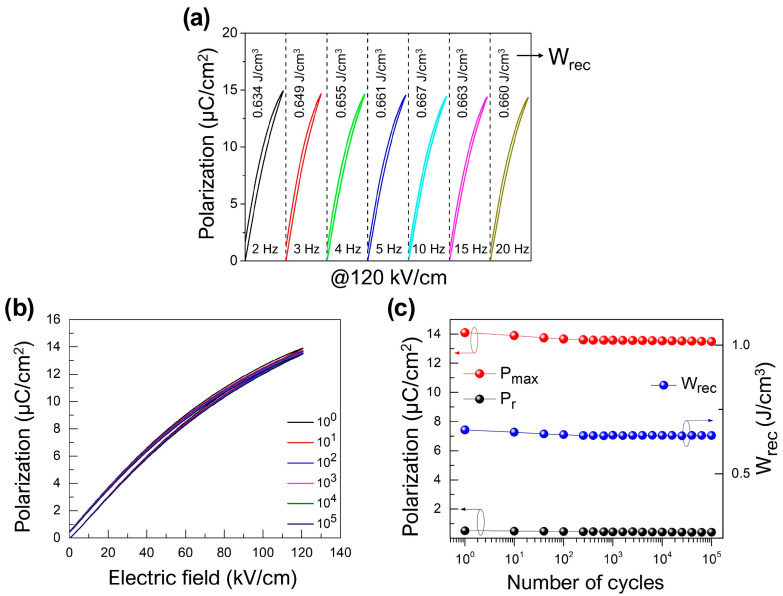
(**a**) Unipolar P-E curves at various frequencies (2–20 Hz) of 0.7BT-0.3(SBT-BMT) specimen under 120 kV/cm field, (**b**) unipolar P-E curves of 0.7BT-0.3(SBT-BMT) ceramic from 1 to 10^5^ cycles at 10 Hz, and (**c**) calculated *P_max_*, *P_r_*, and *W_rec_* as a function of cycle.

Figure 7a shows the room temperature unipolar P-E curves of the 0.7BT-0.3(SBT-BMT) specimen at various frequencies under a 120 kV/cm field. In the frequency range from 2 to 20 Hz, there was no significant change in the P-E curves or energy density values, suggesting a good frequency stability of the energy storage performance. Furthermore, as seen in Figure 7b,c, the 0.7BT-0.3(SBT-BMT) specimen maintained a high *P_max_* and low *P_r_* even after 10^5^ cycles of charge–discharge tests at an electric field of 120 kV/cm. Consequently, *W_rec_* showed a minimal decrease of within 3%, validating the good cyclability of the 0.7BT-0.3(SBT-BMT) specimen. 

## 4. Conclusions

In this study, we investigated the structural, dielectric, ferroelectric, and energy storage properties concerning the variations in x in the (1−x)BT-x(SBT-BMT) system, designed to meet the conditions for a superparaelectric (SPE) state (*T_max_* < *T* < *T_B_*) near room temperature. Analyses from XRD and Raman spectroscopy revealed a shift in the crystal structure from tetragonal to cubic with increasing x content. The relative dielectric permittivity versus temperature curves flattened, and *T_max_* decreased as x increased, indicating enhanced relaxor behavior. Furthermore, except for the x = 0.1 composition, all compositions met the criteria of *T_max_* < *T* < *T_B_* near room temperature, signifying the manifestation of the SPE state in these compositions. A comparative examination of P-E curves across compositions revealed that the x = 0.2 specimen, at temperatures around room temperature, displayed characteristics at the boundary between the relaxor ferroelectric and SPE phases. It exhibited a relatively low SPE state fraction and retained attributes of normal relaxor ferroelectric behavior. On the other hand, specimens with x ≥ 0.3 exhibited slimmer P-E curves with a lower *T_max_* compared to the x = 0.2 specimen, indicating an increased fraction of the SPE state at room temperature. Despite the decline in *P_max_*, the x ≥ 0.3 specimens showed impressive energy storage capabilities, which can be attributed to significantly reduced *P_r_* and elevated *E_max_* values, both stemming from the enhanced SPE state. Particularly, the x = 0.3 (0.7BT-0.3(SBT-BMT)) ceramic exhibited outstanding energy storage characteristics, with *W_rec_* = 1.12 J/cm^3^ and *η* of 94% at 170 kV/cm. Moreover, after 10^5^ charge–discharge cycles at 120 kV/cm, the *W_rec_* only displayed a minimal decrease of within 3%, confirming its good stability. This result highlights the significance of the SPE phase in enhancing energy storage capabilities within relaxor ferroelectric materials, showcasing the potential applicability of the (1−x)BT-x(SBT-BMT) system in high-energy-density storage devices.

## Figures and Tables

**Table 1:** Calculated lattice parameters and unit cell volume of (1−x)BT-x(SBT-BMT) ceramics.

Composition	Space Group	Lattice Parameters	Volume of Unit Cell
x = 0.1	*P*4*mm*	*a* = *b* = 3.99555 ± 0.00003 Å *c* = 4.01797 ± 0.00004 Å *c*/*a* = 1.00561 ± 0.00001	64.145 ± 0.001 Å^3^
x = 0.2	$Pm\bar{3}m$	*a* = *b* = *c* = 3.99556 ± 0.00001 Å *c*/*a* = 1	63.787 ± 0.001 Å^3^
x = 0.3	$Pm\bar{3}m$	*a* = *b* = *c* = 3.98836 ± 0.00001 Å *c*/*a* = 1	63.443 ± 0.001 Å^3^
x = 0.4	$Pm\bar{3}m$	*a* = *b* = *c* = 3.98101 ± 0.00001 Å *c*/*a* = 1	63.093 ± 0.001 Å^3^

**Table 2:** Comparison of the energy storage properties of the 0.7BT-0.3(SBT-BMT) ceramic with other BaTiO_3_-based ceramics.

Composition	Energy Storage Density, *W_rec_* (J/cm^3^)	Efficiency, *η* (%)	Applied Field (kV/cm)	Reference
0.91BaTiO_3_-0.09BiYbO_3_	0.71	82.6	93	[54]
0.85BaTiO_3_-0.15Bi(Mg_1/2_Zr_1/2_)O_3_	1.25	95.4	185	[55]
0.85BaTiO_3_-0.15Bi(Zn_2/3_Nb_1/3_)O_3_	0.79	93.5	131	[56]
0.92(0.65BaTiO_3_-0.35Bi_0.5_Na_0.5_TiO_3_)-0.08SrY_0.5_Nb_0.5_O_3_	1.36	74.3	152	[57]
0.88Ba_0.8_Sr_0.2_TiO_3_-0.12BiTaO_3_	0.526	98	130	[58]
BaTi_0.95_Mg_0.05_O_3_	1.04	89.65	350	[59]
0.93BaTiO_3_-0.07YNbO_4_	0.614	86.8	173	[60]
0.86BaTiO_3_-0.1BiYbO_3_-0.04BiAlO_3_	0.59	97.44	110	[61]
(Ba_0.9_Bi_0.1_)(Ti_0.9_Mg_0.2/3_Ta_0.1/3_)O_3_	5.97	87.4	710	[62]
0.86BaTiO_3_-0.14Bi(Zn_0.5_Ti_0.5_)O_3_	0.81	94	120	[63]
0.7BaTiO_3_-0.3[0.75(Sr_0.88_Bi_0.08_)TiO_3_-0.25Bi(Mg_0.5_Ti_0.5_)O_3_]	1.12	94	170	This study

## Data Availability

Data are contained within the article.

## References

[1] Chu B., Zhou X., Ren K., Neese B., Lin M., Wang Q., Bauer F., Zhang Q.M. (2006). A Dielectric Polymer with High Electric Energy Density and Fast Discharge Speed. Science.

[2] Li Q., Chen L., Gadinski M.R., Zhang S., Zhang G., Li H.U., Iagodkine E., Haque A., Chen L.-Q., Jackson T.N. (2015). Flexible high-temperature dielectric materials from polymer nanocomposites. Nature.

[3] Palneedi H., Peddigari M., Hwang G.-T., Jeong D.-Y., Ryu J. (2018). High-Performance Dielectric Ceramic Films for Energy Storage Capacitors: Progress and Outlook. Adv. Funct. Mater..

[4] Pan H., Li F., Liu Y., Zhang Q., Wang M., Lan S., Zheng Y., Ma J., Gu L., Shen Y. (2019). Ultrahigh–energy density lead-free dielectric films via polymorphic nanodomain design. Science.

[5] Kim J., Saremi S., Acharya M., Velarde G., Parsonnet E., Donahue P., Qualls A., Garcia D., Martin L.W. (2020). Ultrahigh capacitive energy density in ion-bombarded relaxor ferroelectric films. Science.

[6] Yang D., Tian J., Tian S., Yu F., Ren K. (2023). Composition design of BNBT-ST relaxor ferroelectric ceramics in superparaelectric state with ultrahigh energy density. Ceram. Int..

[7] Wang K., Ouyang J., Wuttig M., Zhao Y.-Y., Cheng H., Zhang Y., Su R., Yan J., Zhong X., Zeng F. (2020). Superparaelectric (Ba_0.95_,Sr_0.05_)(Zr_0.2_,Ti_0.8_)O_3_ Ultracapacitors. Adv. Energy Mater..

[8] Pan H., Lan S., Xu S., Zhang Q., Yao H., Liu Y., Meng F., Guo E.-J., Gu L., Yi D. (2021). Ultrahigh energy storage in superparaelectric relaxor ferroelectrics. Science.

[9] Yang L., Kong X., Li F., Hao H., Cheng Z., Liu H., Li J.-F., Zhang S. (2019). Perovskite lead-free dielectrics for energy storage applications. Prog. Mater Sci..

[10] Cho S., Yun C., Kim Y.S., Wang H., Jian J., Zhang W., Huang J., Wang X., Wang H., MacManus-Driscoll J.L. (2018). Strongly enhanced dielectric and energy storage properties in lead-free perovskite titanate thin films by alloying. Nano Energy.

[11] Ogihara H., Randall C.A., Trolier-McKinstry S. (2009). High-Energy Density Capacitors Utilizing 0.7BaTiO_3_–0.3BiScO_3_ Ceramics. J. Am. Ceram. Soc..

[12] Pan H., Ma J., Ma J., Zhang Q., Liu X., Guan B., Gu L., Zhang X., Zhang Y.-J., Li L. (2018). Giant energy density and high efficiency achieved in bismuth ferrite-based film capacitors via domain engineering. Nat. Commun..

[13] Peng B., Zhang Q., Li X., Sun T., Fan H., Ke S., Ye M., Wang Y., Lu W., Niu H. (2015). Giant Electric Energy Density in Epitaxial Lead-Free Thin Films with Coexistence of Ferroelectrics and Antiferroelectrics. Adv. Electron. Mater..

[14] Dong X., Li X., Chen X., Wu J., Zhou H. (2021). Simultaneous enhancement of polarization and breakdown strength in lead-free BaTiO_3_-based ceramics. Chem. Eng. J..

[15] Yuan Q., Li G., Yao F.-Z., Cheng S.-D., Wang Y., Ma R., Mi S.-B., Gu M., Wang K., Li J.-F. (2018). Simultaneously achieved temperature-insensitive high energy density and efficiency in domain engineered BaTiO_3_-Bi(Mg_0.5_Zr_0.5_)O_3_ lead-free relaxor ferroelectrics. Nano Energy.

[16] Yang Z., Du H., Jin L., Hu Q., Wang H., Li Y., Wang J., Gao F., Qu S. (2019). Realizing high comprehensive energy storage performance in lead-free bulk ceramics via designing an unmatched temperature range. J. Mater. Chem. A.

[17] Bell A.J. (1993). Calculations of dielectric properties from the superparaelectric model of relaxors. J. Phys. Condens. Matter.

[18] Li F., Zhang S., Damjanovic D., Chen L.-Q., Shrout T.R. (2018). Ferroelectrics: Local Structural Heterogeneity and Electromechanical Responses of Ferroelectrics: Learning from Relaxor Ferroelectrics (Adv. Funct. Mater. 37/2018). Adv. Funct. Mater..

[19] Bokov A.A., Ye Z.-G. (2012). Dielectric Relaxation in Relaxor Ferroelectrics. J. Adv. Dielectr..

[20] Zhang G.-F., Cao M., Hao H., Liu H. (2013). Energy Storage Characteristics in Sr_(1-1.5x)_Bi_x_TiO_3_ Ceramics. Ferroelectrics.

[21] Li L., Fan P., Wang M., Takesue N., Salamon D., Vtyurin A.N., Zhang Y., Tan H., Nan B., Lu Y. (2021). Review of lead-free Bi-based dielectric ceramics for energy-storage applications. J. Phys. D Appl. Phys..

[22] Zuo C., Yang S., Cao Z., Yu H., Wei X. (2022). Excellent energy storage and hardness performance of Sr_0.7_Bi_0.2_TiO_3_ ceramics fabricated by solution combustion-synthesized nanopowders. Chem. Eng. J..

[23] Ang C., Yu Z., Vilarinho P.M., Baptista J.L. (1998). Bi:SrTiO_3_: A quantum ferroelectric and a relaxor. Phys. Rev. B.

[24] Okhay O., Wu A., Vilarinho P.M., Tkach A. (2011). Dielectric relaxation of Sr_1–1.5x_Bi_x_TiO_3_ sol-gel thin films. J. Appl. Phys..

[25] Zhu X., Shi P., Kang R., Li S., Wang Z., Qiao W., Zhang X., He L., Liu Q., Lou X. (2021). Enhanced energy storage density of Sr_0.7_Bi_x_TiO_3_ lead-free relaxor ceramics via A-site defect and grain size tuning. Chem. Eng. J..

[26] Zhao P., Tang B., Si F., Yang C., Li H., Zhang S. (2020). Novel Ca doped Sr_0.7_Bi_0.2_TiO_3_ lead-free relaxor ferroelectrics with high energy density and efficiency. J. Eur. Ceram. Soc..

[27] Chao M., Liu J., Zeng M., Wang D., Yu H., Yuan Y., Zhang S. (2018). High discharge efficiency of (Sr, Pb, Bi) TiO_3_ relaxor ceramics for energy-storage application. Appl. Phys. Lett..

[28] Kong X., Yang L., Cheng Z., Zhang S. (2020). Bi-modified SrTiO_3_-based ceramics for high-temperature energy storage applications. J. Am. Ceram. Soc..

[29] Yu Z., Ang C., Guo R., Bhalla A.S. (2003). Dielectric properties and tunability of (Sr,Bi)TiO_3_ with MgO additive. Mater. Lett..

[30] Xiong B., Hao H., Zhang S., Liu H., Cao M. (2011). Structure, Dielectric Properties and Temperature Stability of BaTiO_3_–Bi(Mg_1/2_Ti_1/2_)O_3_ Perovskite Solid Solutions. J. Am. Ceram. Soc..

[31] Wu X., Zhao H., Han W., Wang Z., Li F., Li J., Xue W. (2023). Facile preparation and dielectric properties of BaTiO_3_ with different particle sizes and morphologies. RSC Adv..

[32] Yu P., Liu W., Gao P., Shao T., Zhao S., Han Z., Gu X., Zhang J., Wang Y. (2022). Investigation on synthesis of tetragonal BaTiO_3_ nanopowders by a new wet chemical method. J. Mater. Sci. Mater. Electron..

[33] Shannon R.D. (1976). Revised effective ionic radii and systematic studies of interatomic distances in halides and chalcogenides. Acta Crystallogr. Sect. A.

[34] Tkach A., Okhay O. (2021). Comment on “Hole-pinned defect-dipoles induced colossal permittivity in Bi doped SrTiO_3_ ceramics with Sr deficiency”. J. Mater. Sci. Technol..

[35] DiDomenico M., Wemple S.H., Porto S.P.S., Bauman R.P. (1968). Raman Spectrum of Single-Domain BaTiO_3_. Phys. Rev..

[36] Pasuk I., Neațu F., Neațu Ș., Florea M., Istrate C.M., Pintilie I., Pintilie L. (2021). Structural Details of BaTiO_3_ Nano-Powders Deduced from the Anisotropic XRD Peak Broadening. Nanomaterials.

[37] Yadav A.K., Fan H., Yan B., Wang W., Dong W., Wang S. (2021). Structure evolutions with enhanced dielectric permittivity and ferroelectric properties of Ba_(1−x)_(La, Li)_x_TiO_3_ ceramics. J. Mater. Sci. Mater. Electron..

[38] Shi C., Billinge S.J.L., Puma E., Bang S.H., Bean N.J.H., de Sugny J.-C., Gambee R.G., Haskell R.C., Hightower A., Monson T.C. (2018). Barium titanate nanoparticles: Short-range lattice distortions with long-range cubic order. Phys. Rev. B.

[39] Veerapandiyan V.K., Khosravi H.S., Canu G., Feteira A., Buscaglia V., Reichmann K., Deluca M. (2020). B-site vacancy induced Raman scattering in BaTiO_3_-based ferroelectric ceramics. J. Eur. Ceram. Soc..

[40] Pokorný J., Pasha U.M., Ben L., Thakur O.P., Sinclair D.C., Reaney I.M. (2011). Use of Raman spectroscopy to determine the site occupancy of dopants in BaTiO_3_. J. Appl. Phys..

[41] Parsons J.L., Rimai L. (1967). Raman spectrum of BaTiO_3_. Solid State Commun..

[42] Wu Y., Fan Y., Liu N., Peng P., Zhou M., Yan S., Cao F., Dong X., Wang G. (2019). Enhanced energy storage properties in sodium bismuth titanate-based ceramics for dielectric capacitor applications. J. Mater. Chem. C.

[43] Jiang X., Hao H., Yang Y., Zhou E., Zhang S., Wei P., Cao M., Yao Z., Liu H. (2021). Structure and enhanced dielectric temperature stability of BaTiO_3_-based ceramics by Ca ion B site-doping. J. Mater..

[44] Yao G., Wang X., Yang Y., Li L. (2010). Effects of Bi_2_O_3_ and Yb_2_O_3_ on the Curie Temperature in BaTiO_3_-Based Ceramics. J. Am. Ceram. Soc..

[45] Liu J., Ma C., Zhao X., Ren K., Zhang R., Shang F., Du H., Wang Y. (2023). Structure, dielectric, and relaxor properties of BaTiO_3_-modified high-entropy (Bi_0.2_Na_0.2_K_0.2_Ba_0.2_Ca_0.2_)TiO_3_ ceramics for energy storage applications. J. Alloys Compd..

[46] Hu Q., Tian Y., Zhu Q., Bian J., Jin L., Du H., Alikin D.O., Shur V.Y., Feng Y., Xu Z. (2020). Achieve ultrahigh energy storage performance in BaTiO_3_–Bi(Mg_1/2_Ti_1/2_)O_3_ relaxor ferroelectric ceramics via nano-scale polarization mismatch and reconstruction. Nano Energy.

[47] Zuo J., Yang H., Chen J., Li C. (2023). Highly-reliable dielectric capacitors with excellent comprehensive energy-storage properties using Bi_0.5_Na_0.5_TiO_3_-based relaxor ferroelectric ceramics. J. Eur. Ceram. Soc..

[48] Hu D., Pan Z., Zhang X., Ye H., He Z., Wang M., Xing S., Zhai J., Fu Q., Liu J. (2020). Greatly enhanced discharge energy density and efficiency of novel relaxation ferroelectric BNT–BKT-based ceramics. J. Mater. Chem. C.

[49] Huang N., Liu H., Hao H., Yao Z., Cao M., Xie J. (2019). Energy storage properties of MgO-doped 0.5Bi_0·5_Na_0·5_TiO_3_-0.5SrTiO_3_ ceramics. Ceram. Int..

[50] Brown E., Ma C., Acharya J., Ma B., Wu J., Li J. (2014). Controlling Dielectric and Relaxor-Ferroelectric Properties for Energy Storage by Tuning Pb_0.92_La_0.08_Zr_0.52_Ti_0.48_O_3_ Film Thickness. ACS Appl. Mater. Interfaces.

[51] Wu J., Mahajan A., Riekehr L., Zhang H., Yang B., Meng N., Zhang Z., Yan H. (2018). Perovskite Sr_x_(Bi_1−x_Na_0.97−x_Li_0.03_)_0.5_TiO_3_ ceramics with polar nano regions for high power energy storage. Nano Energy.

[52] Petzelt J. (2010). Dielectric Grain-Size Effect in High-Permittivity Ceramics. Ferroelectrics.

[53] Huamán J.L.C., Rivera V.A.G., Pinto A.H., Marega E., Huamán J.L.C., Rivera V.A.G. (2023). 11-Multiferroic perovskite ceramics: Properties and applications. Perovskite Ceramics.

[54] Shen Z., Wang X., Luo B., Li L. (2015). BaTiO_3_–BiYbO_3_ perovskite materials for energy storage applications. J. Mater. Chem. A.

[55] Jiang X., Hao H., Zhang S., Lv J., Cao M., Yao Z., Liu H. (2019). Enhanced energy storage and fast discharge properties of BaTiO_3_ based ceramics modified by Bi(Mg_1/2_Zr_1/2_)O_3_. J. Eur. Ceram. Soc..

[56] Wu L., Wang X., Li L. (2016). Lead-free BaTiO_3_–Bi(Zn_2/3_Nb_1/3_)O_3_ weakly coupled relaxor ferroelectric materials for energy storage. RSC Adv..

[57] Liu X., Yang H., Yan F., Qin Y., Lin Y., Wang T. (2019). Enhanced energy storage properties of BaTiO_3_-Bi_0.5_Na_0.5_TiO_3_ lead-free ceramics modified by SrY_0.5_Nb_0.5_O_3_. J. Alloys Compd..

[58] Zhao H., Duan X., Yu T., Xu D., Zhao W. (2023). Enhanced energy storage efficiency of Ba_0.8_Sr_0.2_TiO_3_ ceramics modified by BiTaO_3_. Solid State Commun..

[59] Borkar S.N., Deshpande V.K. (2022). Effect of Mg substitution on microstructural, dielectric, ferroelectric and energy storage properties of BaTiO_3_. Phys. B Condens. Matter..

[60] Dong X., Chen H., Wei M., Wu K., Zhang J. (2018). Structure, dielectric and energy storage properties of BaTiO_3_ ceramics doped with YNbO_4_. J. Alloys Compd..

[61] Yi X., Ji C., Chen G., Yang H., Yong H., Fu C., Cai W., Gao R., Fan T., Wang Z. (2020). Effects of Sintering Method and BiAlO_3_ Dopant on Dielectric Relaxation and Energy Storage Properties of BaTiO_3_–BiYbO_3_ Ceramics. Phys. Status Solidi A.

[62] Yin M., Zhang Y., Bai H.-R., Li P., Li Y.-C., Han W.-F., Hao J.-G., Li W., Wang C.-M., Fu P. (2024). Preeminent energy storage properties and superior stability of (Ba_(1–x)_Bi_x_)(Ti_(1–x)_Mg_2x/3_Ta_x/3_)O_3_ relaxor ferroelectric ceramics via elongated rod-shaped grains and domain structural regulation. J. Mater. Sci. Technol..

[63] Zhao X., Zhou Z., Liang R., Liu F., Dong X. (2017). High-energy storage performance in lead-free (1-x)BaTiO_3_-xBi(Zn_0.5_Ti_0.5_)O_3_ relaxor ceramics for temperature stability applications. Ceram. Int..

